# Toxicity at the Edge of Life: A Review on Cyanobacterial Toxins from Extreme Environments

**DOI:** 10.3390/md15070233

**Published:** 2017-07-24

**Authors:** Samuel Cirés, María Cristina Casero, Antonio Quesada

**Affiliations:** 1 Departamento de Biología, Darwin, 2, Universidad Autónoma de Madrid, 28049 Madrid, Spain; antonio.quesada@uam.es; 2 Museo Nacional de Ciencias Naturales, MNCN-CSIC, Calle Serrano 115, 28006 Madrid, Spain; mcristina.casero@mncn.csic.es

**Keywords:** anatoxin-a, cylindrospermopsin, microcystin, nodularin, extremophiles, Arctic, Antarctica, hot deserts, hypersaline, alkaline lakes

## Abstract

Cyanotoxins are secondary metabolites produced by cyanobacteria, of varied chemical nature and toxic effects. Although cyanobacteria thrive in all kinds of ecosystems on Earth even under very harsh conditions, current knowledge on cyanotoxin distribution is almost restricted to freshwaters from temperate latitudes. In this review, we bring to the forefront the presence of cyanotoxins in extreme environments. Cyanotoxins have been reported especially in polar deserts (both from the Arctic and Antarctica) and alkaline lakes, but also in hot deserts, hypersaline environments, and hot springs. Cyanotoxins detected in these ecosystems include neurotoxins—anatoxin-a, anatoxin-a (S), paralytic shellfish toxins, β-methylaminopropionic acid, *N*-(2-aminoethyl) glycine and 2,4-diaminobutyric acid- and hepatotoxins –cylindrospermopsins, microcystins and nodularins—with microcystins being the most frequently reported. Toxin production there has been linked to at least eleven cyanobacterial genera yet only three of these (*Arthrospira*, *Synechococcus* and *Oscillatoria*) have been confirmed as producers in culture. Beyond a comprehensive analysis of cyanotoxin presence in each of the extreme environments, this review also identifies the main knowledge gaps to overcome (e.g., scarcity of isolates and –omics data, among others) toward an initial assessment of ecological and human health risks in these amazing ecosystems developing at the very edge of life.

## 1. Introduction

Cyanobacteria are photosynthetic prokaryotes that play a significant role in the production of organic matter and oxygen [1], being the most important component of photoautotrophic microflora in terms of total biomass and productivity. They are widely distributed in both aquatic and terrestrial ecosystems and are capable of thriving in some of the most extreme environments on Earth, varying from the hyper-arid, cold Antarctic Dry Valleys to the hot, dry Atacama Desert [2,3]. Despite this worldwide distribution, most of the research has focused on lentic freshwater ecosystems (lakes, ponds and reservoirs) from temperate latitudes, due to the frequent development of mass proliferations (blooms) of planktonic cyanobacteria in those water bodies [4].

Besides their ecological plasticity, cyanobacteria are characterized by a very active secondary metabolism, that is, a set of processes apparently not involved in primary metabolism (e.g., photosynthesis, respiration). As a product of such, cyanobacteria are the source of more than 300 bioactive compounds [5], many of which are yet of unknown function. Among those secondary metabolites, cyanotoxins have received most of the research attention so far. Cyanotoxins are a group of chemically diverse substances (e.g., alkaloids, cyclic peptides, non proteic amino acids) with documented adverse effects on humans, animals, plants and eukaryotic microbes [6]. According to their effects, cyanotoxins can be classified into hepatotoxins (e.g., the cyclic peptides microcystins -MCs- and nodularin –NOD-), neurotoxins (e.g., the alkaloids anatoxin-a, anatoxin-a (S), and paralytic shellfish toxins –PSTs- or saxitoxins) and cytotoxins/dermatotoxins (e.g., the alkaloids cylindrospermopsin –CYN- and lyngbyatoxin). To date, about 40 cyanobacterial genera have been described as potential cyanotoxin producers [7], that is, those taxa for which at least one strain has been confirmed as cyanotoxin producer worldwide.

Cyanotoxins represent a serious threat to water uses of affected bodies worldwide, including drinking water, recreational activities and irrigation. As such, there is vast scientific literature on the ecology and fate of cyanotoxins in temperate freshwater ecosystems and some coastal areas, including also a number of documented cases of animal and human intoxications [6,8]. In contrast, the scarcity of information on the presence of cyanotoxins outside those ecosystems is striking. Given the ecological plasticity of cyanobacteria and their ability to survive under harsh and extreme conditions of temperature, desiccation, salinity, and light irradiation [9] we could hypothesize that potentially toxic cyanobacteria are much more widely distributed than currently thought. If this were the case, cyanotoxicity could be considered a global phenomenon not restricted to just some specific niches.

In this context, the present review provides an outlook on the presence and ecology of cyanotoxins in extreme environments. We will focus on those ecosystems for which cyanotoxin reports are available in the literature: polar deserts (Arctic and Antarctica), hot deserts, alkaline lakes, hot springs and hypersaline environments. In a first section, we will summarize current knowledge on the environmental ranges for cyanotoxin biosynthesis, according to data from laboratory studies. Then, in separate sections we will show an overview on cyanotoxin reports for each of the ecosystems, as well as on the cyanobacteria proposed as cyanotoxin producers in those environments. Finally, we will provide some conclusions and highlight the main challenges to advance in the exciting field of cyanotoxicity at the edges of life.

## 2. Cyanotoxins and Environmental Factors

During the last three decades, a number of studies have tested the influence of environmental factors on cyanotoxins production in laboratory strains of cyanobacteria (Table 1). As a whole, cyanotoxin production has been confirmed in lab cultures under moderately broad environmental ranges of temperature (10–35 °C), light irradiance (1–340 µmol photons m^−2^·s^−1^) and pH (7–14). Studies on salinity have ranged up to near-marine conditions (35‰). In our opinion, the lack of data outside these ranges can be hypothetically attributed to the scarcity of toxic cultures from more extreme habitats (as we will explain in further sections) rather than to the fact that cyanotoxins cannot be produced outside these environmental ranges.

Most of the work available concerns MCs, but there is also information on ATX, CYN, PSTs and NOD. However, as far as we are aware, other toxins that are present in extreme environments have not been investigated in relation with their environmental regulation in lab cultures. Those include the neurotoxic alkaloid anatoxin-a (S), and the neurotoxic aminoacids β-methylaminopropionic acid (BMAA), N-(2-aminoethyl) glycine (AEG) and 2,4-diaminobutyric acid (DAB). These compounds are characterized by the complexity of their analytical determination, the lack of reliable commercial standards and/or their apparent instability [10], reasons that may be behind the scarcity of data on such toxins.

One of the main knowledge gaps concerns the low number of genera tested, which are restricted to the most widely distributed and/or easily cultured organisms. As a consequence, current information is almost entirely limited to the planktonic bloom-forming genera commonly found in freshwaters, such as the chroococcalean *Microcystis*, the nostocalean *Anabaena, Aphanizomenon/Cuspidothrix/Chrysosporum*, *Cylindrospermopsis* and *Raphidiopsis*, and the oscillatorian *Planktothrix*. Data on planktonic genera from non-freshwater environments are limited to the brackish-water thriving *Nodularia*. Strikingly, there are very few studies on benthic cyanotoxins producers related to environmental factors in laboratories. Those available focused on the nostocalean genus *Nostoc* and the oscillatorian *Phormidium* and *Oscillatoria*. In spite of the relatively important amount of publications on environmental regulation of cyanotoxins production, it seems fairly insufficient that overall results are limited to 11 cyanobacterial genera (Table 1) out of the at least 40 cyanotoxin-producing genera reported worldwide to date [7].

In general, in studies summarized in Table 1, cyanotoxin production seemed to occur as long as net growth was present. However, some of the experiments showed that cyanotoxin production may drop off suddenly (or remain below detection limits) at conditions close to the environmental extremes, right before the growth rates suffer a detectable decrease. For instance, Cirés et al. [17] observed a drastic 25-fold decrease in CYN production by cultures of *Chrysosporum ovalisporum* (basionym *Aphanizomenon ovalisporum*) when temperature was increased from 30 to 35 °C, while growth did not decrease at 35 °C but suffered a very sudden decay at 40 °C. Similarly, CYN production was completely inhibited at 35 °C in *Cylindrospermopsis raciborskii* [18], which could be attributed to the loss of activity in the amidinotransferase CyrA involved in CYN biosynthesis [19]. In another interesting experiment on *Microcystis* cultures subjected to various salinities, Tonk et al. [12] observed that MC was no longer detected at salinities over 10‰, yet *Microcystis* was still able to grow at a salinity of up to 17‰. All in all these findings suggest that cyanotoxin synthesis may in some cases cease in response to environmental extremes slightly before growth or primary metabolism are conspicuously affected. However, the lack of standardization in methods used in the “black-box” experiments summarized in Table 1 precludes from extracting solid conclusions in this regard, since the apparent lack of toxin at the extremes may just be caused by a low (yet present) toxin concentration below detection limits of the quantification techniques utilized.

In this sense, molecular studies on the expression of cyanotoxin biosynthesis genes and/or the activity of the encoded enzymes seem a much more trustworthy option to understand actual regulation of toxin production by environmental factors. Unfortunately, studies using -omics on cyanotoxin-producing cultures subjected to environmental factors are rather scarce, and currently they are mostly limited to the transcription analysis of some genes in very few cyanobacterial genera [10]. Proteomics and metabolomics, on the other hand, are restricted to the investigations in saxitoxin-producing *Anabaena circinalis* by [20]. Keeping in mind the impossibility of generalization and the need for more data, molecular works have not yet found unequivocal “switch-on/switch-off” mechanisms for the expression of genes involved in cyanotoxin synthesis. Until those mechanisms are found, we may reasonably argue that the environmental limits for cyanotoxin production are those for the growth of the producing organisms. This is coherent with the occurrence of cyanotoxins already reported in extreme environments, under environmental conditions clearly outside the ranges so far studied in the laboratory as we will see in further sections.

## 3. Polar Deserts

### 3.1. Ecosystem Description

The cryosphere, defined as the ensemble of all ice-containing environments on planet Earth, is now recognized to be a living system with a diversity of microbial life of great interest. Snow is the most conspicuous part of this system providing a habitat for microbial communities in high latitudes [21]. The so-called cryoconite communities of cyanobacteria occur in melt-holes on glaciers in the Arctic and Antarctica. Several other types of extreme low temperature habitats have now been described including lake-ice [22], streams beneath glaciers [23], and the junction between ice crystals in the Antarctic ice cap [24].

These environments are dominated by strong gradients in temperature (from −10 to −2 °C), salinity (from 35‰ to 150‰), space and irradiation (<0.1% UV radiation to 1–5% UV radiation). All these properties and the morphology of the system are highly variable, being ultimately governed by air temperature and snow cover. Large seasonal and diurnal differences in ice properties occur, with change in salinity regarded as the dominant factor in external chemistry [25]. Hyperoxic brines depleted in CO_2_, high concentrations of dissolved organic matter, low concentrations of nutrients, high ammonia concentrations and elevated pH values are the consequence of biological activity within the confined system [26].

In the absence of other photoautotrophic organisms, it is accepted that cyanobacteria are largely responsible for providing the most important exosystem services, and that cyanobacterial autotrophy supports substantial and diverse populations of heterotrophic microorganisms together with smaller numbers of organisms in higher trophic levels [27,28,29]. It is widely accepted that cyanobacteria are responsible for sustaining crucial ecosystem services in high latitude Polar Regions.

The distribution of cyanobacteria in polar soil may respond to environmental gradients, their abundance has been shown to be linked to latitudinal gradients, with the highest diversity of cyanobacteria between 70 and 80 °S [30].

### 3.2. Cyanotoxins

Cyanotoxin presence has been reported in benthic communities of both Arctic and Antarctica (Table 2), comprising cyanobacterial-dominated mats/biocrusts developing in freshwaters (e.g., lakes—cryogenic lakes, large cirque lakes-; ponds—small kettle ponds, meltwater ponds-; meltwater runoff and, small streams, lagoon channels) [31,32,33,34,35], brackish ponds [34], hydroterrestrial environments and wet soils [33,36,37] (Table 2) as well as symbiotic associations (cyanolichens) between cyanobacteria and lichens in the Arctic [38,39]. Toxins detected include the hepatotoxins MC (in both the Arctic and Antarctica) [32,33,35,36,37,38,39,40] and NOD (in Antarctica) [34], the cytotoxin CYN (in Antarctica) [31], and the neurotoxins ATX [37] and PST [33] in the Arctic (Table 2). In those investigations, about 20–96% of the screened samples contained cyanotoxins which indicates high variability depending on the study. This also seems to be in the broad range of 4–90% cyanotoxin positive samples found in cyanobacterial blooms worldwide [8,41], although it should be interpreted cautiously given the very low number of studies and samples in polar ecosystems.

Beyond benthic samples, cyanotoxin reports in pelagic samples are currently restricted to the detection of MCs in 18 lakes in Southwestern Greenland [40]. In that work, the authors analyzed total MCs from whole-water lake samples by ELISA and reported very low concentrations (0.005–0.4 µg MCs/L). Unfortunately, no data were provided about the taxa present in the lakes.

MCs are the most frequently reported toxin in both poles and also those with the maximum content measured with 11–16 µg MC/g dry weight biomass, with lower 0.02–0.6 µg toxin/g dry weight biomass reported for ATX, CYN or PST. No concentrations have been published for NOD as far as we are aware. The maximum cyanotoxin contents reported in Arctic and Antarctic biomass samples are clearly lower than maxima measured in phytoplankton biomass from temperate or tropical/subtropical freshwaters (e.g., see in Table 3 the 223–4593 µg MC/g dry weight measured in Kenyan Lakes) reaching up to 20,000 µg MC/g DW in surface “scums” [8].

Further research is needed to determine whether this apparently lower production is mainly due to an actual reduced toxin biosynthesis by polar strains and/or under harsh polar environmental conditions or just to a low proportion of toxin-producing strains in those microbial mats. In this sense, studies focused on the influence of environmental factors in toxin production by polar communities are nearly absent, with the exception of findings by Kleinteich et al. [32] indicating a positive influence of temperature increases (from 4 to 8–16 °C, potentially reached during summer months in polar regions at the present warming rate) on MC production by Arctic and Antarctic cyanobacterial mats. Studying lichens, Kaasalainen et al. [38] observed that the percentage of toxinogenic (*mcyE*-positive) specimens was higher (8–9%) in boreal and arctic climates than in tropical rain forests (3%). These studies raise interesting questions on the influence of biogeography and environmental shifts (e.g., climate change) on toxin production in polar systems which need to be addressed by further research in the near future.

According to mass spectrometry analyses, MC congeners reported in the poles include MC-LR, MC-RR, MC-FR and variants thereof, many of which are characterized by rather uncommon amino acid substitutions [33,36,42]. Puddick et al. [42] using stereochemical analysis of amino acids and thiol derivatization, found that MCs from McMurdo, Antarctica, contained the rare substitution of the position-1 d-alanine for glycine, the acetyl desmethyl modification of the position-5 Adda moiety (3S-amino-9S-methoxy-2S,6,8S-trimethyl-10-phenyldeca-4E,6E-dienoic acid) and the presence of dehydrobutyrine in position-7 instead of the commonly observed N-methyl dehydroalanine. In the Arctic, Kleinteich et al. [33] identified the variant [Asp3, ADMADDA5, Dhb7] MC-RR which before that study had only been reported in a *Nostoc* sp. strain from a UK lake. Kaasalainen et al. [38] also found [Leu^1^] MC-LR (Leu in positions one and two, and Arg in position four) in cyanolichens from Jehkats, Lapland, an MC variant again infrequently found among free-living cyanobacteria. Regarding cyanotoxins other than MCs, Kleinteich et al. [31] detected the common CYN variant deoxy-CYN. It remains unknown which of the 56 PST variants identified to date [43] are produced by polar cyanobacteria. Indeed, in the only study on the topic, despite detecting PSTs by the enzyme-linked immunosorbent assay (ELISA), no PST chemical variants could be identified in subsequent HPLC-FD analyses [33].

To date there is no confirmation of cyanotoxin production in unicyanobacterial cultures isolated from polar ecosystems; therefore, the identification of cyanotoxin producers remains just putative (Table 2). This may be attributed in part to the difficulty of maintaining polar cyanobacterial strains under laboratory conditions. Additionally, molecular detection and sequencing of genes involved in toxin biosynthesis have been carried out only in 5 out of the 10 field studies shown in Table 2. Wood et al. [36] obtained a clone library for *mcyE* gene (MC synthetase) in mats from McMurdo Dry Valleys, identifying *Nostoc* sp. as the most likely MC producer (93% *mcyE* sequence similarity with *Nostoc* sp. 152, 364 bp). In the Arctic (Lapland and Svalbard), *mcyE* phylogeny of cyanolichens also pointed out to *Nostoc* spp. genotypes as the MC producer in associations with lichen genera *Peltigera*, *Nephroma* and *Protopannaria* [38,39]. *Nostoc* spp. have also been proposed as potential MC producers in Adelaide Island, Antarctica [33] and Baffin Island, Canadian Arctic [32] but without molecular confirmation by toxin-biosynthesis gene sequencing.

Members of the genera *Scytonema* sp. and *Lyngbya* sp. have been labelled as the likely candidates for PST production in Baffin Island (Canadian Artic) according to sequences of *sxtA* (polyketide synthase) from field samples, despite the relatively low similarity to NCBI GenBank sequences (*Scytonema* cf. *crispum*, 97%, *Lyngbya wollei*, 95%) did not allow an unequivocal identification at species level [33]. That same study also detected mRNA of *sxtA* gene, confirming that the gene was being transcribed under field conditions. It is important to remark that *sxtA* gene presence alone is not sufficient proof for PST production, since this gene has been detected in a number of non-PST producing cyanobacterial strains of nostocalean genera *Anabaena*, *Anabaenopsis*, *Aphanizomenon* and *Cuspidothrix* [44]. Therefore screening additional saxitoxin-biosynthesis genes other than *sxtA*, (e.g., *sxtH*, *sxtI*, *sxtX*, see [44]) seems recommendable for further molecular studies on polar cyanobacterial samples. 

CYN production in Adelaide Island, Antarctica, was linked to the genus *Oscillatoria*, as clones of CYN-biosynthesis gene amplicons yielded a sequence similarity from 93% (*cyrJ* gene, 584 bp fragment) to 96% (single 2200 bp amplicon including partial *cyrAB* genes) with published sequences of CYN-producing *Oscillatoria* sp. PCC6506 [45].

In short, current molecular evidence points out to benthic filamentous cyanobacteria of the orders Nostocales (genera *Nostoc, Scytonema*) and Oscillatoriales (genus *Oscillatoria*) as main candidates for cyanotoxin production in polar ecosystems. This is also in line with the fact that no uni-cyanobacterial culture of benthic toxin-producing Chroococcales has been established to date worldwide [46]. The actual toxin-producing capacity of Chroococcales and of filamentous genera (e.g., *Phormidium*, *Lyngbya, Anabaena*) present in the cyanotoxin-positive polar samples and described as cyanotoxin producers elsewhere, still remains to be disentangled by future research.

## 4. Hot Deserts

### 4.1. Ecosystem Description

There is no unanimous consensus on what constitutes a ‘desert’, but a number of factors are synonymous with these environments. In hot deserts, the combined effects of temperature fluctuations and aridity lead to unique adaptations in desert species [47].

These strong ranges in temperature may vary from 5 to 40 °C in the Arabian Desert and from −20 to 30 °C in the Gobi Desert. In relation to the annual precipitation, strong differences can be observed along the different deserts related to their geographical location, from 300 to 500 mm/year in the semiarid Tanami Desert, to 0–20 mm/year in the hyperarid Atacama Desert.

Cyanobacteria are well represented in a range of hot edaphic communities being particularly important in oligotrophic arid environments due to their implication in the key biogeochemical cycling processes and stress response [48].

### 4.2. Cyanotoxins

The scarce field studies in hot deserts depict the presence of the hepatotoxins MCs and the neurotoxins ATX(s), BMAA and its isomers (DAB and AEG) [49,50]. Positive samples included biocrusts from Qatar desert -containing MC, ATX(s) and/or BMAA and isomers- and phytoplankton samples from shallow springs located at oasis complexes in Gobi desert, Mongolia, containing BMAA (Table 3).

The toxin content reported for desert biomass is very low (<0.1 µg/g DW for MCs and ATX(s); and 0.7–7.7 µg/g for BMAA isomers) (Table 3). Furthermore, in the case of BMAA and its isomers, the controversy about the lack of accuracy of current analytical mass spectrometry methods for BMAA [59], raises questions on the reported values suggesting the need for further evidence from additional studies. This is especially relevant considering that some authors have established a causative link between BMAA from desert cyanobacteria and the sporadic appearance of the neurodegenerative disease ALS (amyotrophic lateral sclerosis) among Gulf War veterans [51].

MC-LR was the only MC variant unequivocally identified by HPLC-PDA in 5 cyanobacterial samples taken from wadis—dry, ephemeral river beds—and sabkha—supertidal salt flats—in Qatar [50], although spectral matching and retention times also suggested the presence of additional MC congeners yet not identified. Additionally, the MC-biosynthesis gene mcyD was detected (but not sequenced) in 3 out of the 5 MC-positive samples. This same study affirmed the presence of ATX (S) indicated by acetylcholine esterase inhibition assay, but no structural confirmation of the molecule is provided in the paper. The samples were also screened for ATX and CYN by HPLC-PDA but none of those toxins was found.

In general, the lack of cultures and molecular studies does not allow solid conclusions on the identity of cyanotoxin producers in deserts. In any case, some of the *Phormidium/Microcoleus* morphotypes usually dominating desert crusts may be feasible candidates for ATX or MC production [50] given that the oscillatorial genus *Phormidium* has been widely reported as ATX and MC producer in benthic mats from temperate regions [46].

## 5. Alkaline Lakes

### 5.1. Ecosystem Description

Alkaline environments depend on a continuous process, either microbial or chemical, to maintain an alkaline pH and counteract the buffering effect of the HCO_3_^−^/CO_3_^2−^ system, which at a macroscale tend to maintain a more neutral or acidic pH. Soda lakes are highly alkaline extreme environments that form in closed drainage basins exposed to high evaporation rates. Because of the scarcity of Mg^2+^ and Ca^2+^ in the water chemistry, the lakes become enriched in CO_3_^2−^ and Cl^−^, with pHs in the range 8 to >12. They represent one of the most stable high-pH environments on Earth [60]. Many soda lakes experience massive seasonal or permanent microbial blooms often resulting in distinct coloration of the lake water [61]. Primary production from cyanobacteria in these habitats is very high and supports a diverse alkaliphilic microbial community [62].

### 5.2. Cyanotoxins

Alkaline lakes (pH 9–12) are, together with polar ecosystems, those with a highest number of cyanotoxin reports among extreme environments. However, most cyanotoxin data originate from a relatively restricted geographical area: the Eastern Rift Valley lakes, in Kenya and Tanzania [54,55,63].

ATX and MCs (MC-LR, MC-RR, MC-YR, MC-LA and MC–LF) were detected in phytoplankton populations of 4 Kenyan lakes (Bogoria, Nakuru, Simbi and Sonachi) under extreme water pH of 9.8–10.6. HPLC-PDA measurements evidenced moderate to very high toxin concentrations reaching maxima of 4593 µg equivalents MC-LR/g DW and 223 µg ATX/g DW in *Arthrospira fusiformis*-dominated blooms at Lake Nakuru. Interestingly, toxin production by *A. fusiformis* was confirmed in 3 cultured strains, including 2 *A. fusiformis* strains simultaneously producing MC and ATX (strain AB2002/10 from Lake Bogoria; AB2002/02 from Lake Sonachi) [54,55] and one ATX-producing strain from Lake Nakuru (AB2002/04) [54].

The isolation of toxic *A. fusiformis* strains also shed light on the dramatic history of massive lesser flamingo deaths in Rift Valley Lakes, for which *Arthrospira* biomass is known as one of their main dietary sources (i.e., and adult lesser flamingo consumes about 72 g cyanobacterial biomass/day). Indeed, Krienitz et al. [54] detected MC and ATX in liver and stomach/intestine tissues from lesser flamingos dead at Lakes Bogoria and Nakuru in 2001–2003, in concentrations of up to 0.9 µg MC/g fresh weight tissue and 5.8 µg ATX/g fresh weight tissue. MCs were also linked to the death of lesser flamingos in the Tanzanian alkaline lakes Manyara and Momella, concurrent with up to 1.1 µg MC/g DW in blooms containing *A. fusiformis* but also *Anabaena*, *Nostoc* and *Oscillatoria* (unpublished data from Nonga and Kaaya, see [63]). More recently, Metcalf et al. [64] found trace amounts of BMAA in extracts from Lake Nakuru flamingo feathers, with DAB also present at concentrations between 3.5 and 8.5 g/g dry weight in feathers from Lake Nakuru and Bogoria [64]. Whether BMAA and its isomers are involved in flamingo intoxications is still to be further validated, given the analytical concerns explained in previous sections.

Importantly, toxins from *Arthrospira* in tropical and subtropical lakes may pose an actual threat to human beings. Given that *Arthrospira/Spirulina* was (wrongly) regarded as non-toxic [65], several members of this genus are widely used in mass culture as a source of food, animal feed, and specific chemicals in subtropical and tropical countries [66] with the consequent possibility of intoxications if part of the biomass contains a toxin producing strain or rests of it.

Regarding cyanotoxins beyond African lakes, the hepatotoxin NOD was detected by ELISA in concentrations of 0.3–4.8 µg/L in the water of alkaline Lake Burdur in Turkey, associated to a *Nodularia spumigena* bloom under brackish (20‰ salinity) high pH (9.1) water conditions [56].

## 6. Hypersaline Environments

### 6.1. Ecosystem Description

Hypersaline habitats near or above 150‰ salinity harbor a rich biota adapted to high salinity [67,68]. These kinds of environments have limited microbial diversity due to the combined effects of several factors, including high salt concentrations, temperatures and pH, and low nutrient and oxygen availability [69]. Halophilic microorganisms are classically categorized on the basis of their optimal growth in different salt concentrations, extreme halophiles (above 15% NaCl; equivalent to 150‰ salinity) and moderate halophiles (3–15% NaCl; 30–150‰ salinity).

Cyanobacteria are the main primary producers in inland hypersaline lakes, as well as in coastal hypersaline lagoons, saltern evaporation ponds, saline springs and other environments with salt concentrations exceeding that of seawater (35‰). At highest salt concentrations (from 250‰ up to NaCl saturation), cyanobacteria are rarely found but there are some exceptions, halite evaporates (100% NaCl) in the Atacama Desert, Chile, that contains viable and active cyanobacteria *Chroococidiopsis* like [3] and evaporate crusts with cyanobacteria as well [70].

### 6.2. Cyanotoxins

Despite cyanobacteria are widespread components of high-salt environments (70–350‰ salinity) such as hypersaline lakes, sulphur springs, hypersaline lagoons and salt flats, and man-made salterns, there are few published data on the cyanotoxin production by the communities of halophilic cyanobacteria. Halophilic cyanobacterial communities include the major species *Aphanothece halophytica-Euhalothece-Halothece* Group, *Microcoleus (Coleofasciculus) chthonoplastes*, *Halospirulina tapeticola* and *Halomicronema excentricum,* [71] not usually regarded as cyanotoxin producers, but also less dominating taxa (e.g., *Nodularia, Lyngbya, Phormidium, Oscillatoria*) [71] that have been described as cyanotoxin-producing genera in studies performed in non-saline environments.

Nearly all reports on cyanotoxin production in saline-hypersaline environments (salinity over that of sea water, 35‰ salinity) originate from some of the Rift Valley alkaline lakes described in the previous Section 5.1, i.e., Kenyan lakes Bogoria (50‰ salinity) and Nakuru (10–120‰ salinity) and Tanzanian Lake Manyara (up to 40‰ salinity) [64,65]. Beyond those waterbodies, MC production has been reported only in one *Aphanothece halophytica* strain isolated from non-alkaline Solar Lake [72], a shallow small hypersaline (~180‰) lake on the shore of the Sinai coast, Egypt. Toxin production by *Aphanothece halophytica* was estimated in 3.99 µg eq. MC-LR/g DW by ELISA, and a chromatographic peak with retention time close to that of MC-LR was observed in HPLC-PDA analyses. The authors also linked those findings to the death of mice after 4 h post intraperitoneal injection of methanolic cyanobacterial extracts [72].

The Great Salt Lake is a large lake in Utah, USA, that contains differentiated areas with fluctuating salinities from freshwater to brackish (10–30‰ salinity) and even hypersaline (up to 270‰ salinity). Some portions of the Great Salt Lake such as Farmington Bay, host massive blooms of 100,000–1,500,000 cells/mL of the potentially NOD-producing cyanobacteria *Nodularia spumigena*, mostly in brackish to saline conditions (7–49‰ salinity) [73]. Only certain cultures of *N. spumigena* are able to tolerate salinities of 59‰ salinity especially given that the upper limit of N_2_ fixation for this species has been set at a salinity of 70‰ [71]. ELISA-based NOD analyses performed in Farmington Bay measured 0.2–69.4 µg NOD/L in samples dominated by *Nodularia spumigena* [73]. However, only one out of the five sampling dates could be considered true saline water (18 June 2012, 35–49‰ salinity), whereas most of the toxic samples were recorded in brackish water samples (1–29‰ salinity), such as the maximum 69 µg NOD/L measured in June 2013 coinciding with 393,026 cells *Nodularia*/mL at a salinity of 15‰ [73].

## 7. Hot Springs

### 7.1. Ecosystem Description

Geothermal springs are located non-continuously in all continents, being mainly linked to recent volcanic activity [74]. These hydrothermal waters are intermediate in concentration between dilute inflow and lake waters. The springs are often divided into warm springs and hot springs [75], the former typically situated on fault line and fault intersection sites away from the lake shore whose temperature range is from 34 to 48 °C, while some hot springs can reach temperatures ranging from 64 to 98.5 °C such as the Mwanesis-Kwaipopei-Losaramat group, in Kenia [76]. Hot springs have been of utmost importance because of the unique thermophilic properties of the organisms thriving in these niches.

Temperature and pH in combination with availability of combined nitrogen, phosphorus and other nutrients or concentration of free sulfide determines the distribution of thermophilic cyanobacteria. These organisms are not observed above 76 °C and below a pH of about 4.0. In general, their diversity is limited below pH 6 [77].

### 7.2. Cyanotoxins

MC-containing mats have been reported from hot springs at Jazan, Saudi Arabia, and in the shore of Lake Bogoria, Kenya. The latter mats also contained ATX (Table 3).

Hot springs on the shore of Lake Bogoria comprise a series of boiling springs and fumaroles discharging water characterized not just by high temperature (35–100 °C), but also by elevated values of pH (9.0), conductivity (6410 µS/cm) and salinity (3.5X sea water salinity) [57]. These hot springs host highly diverse cyanobacterial mats dominated by *Phormidium terebriformis*, *Oscillatoria willei*, *Spirulina subsalsa* and *Synechococcus bigranulatus*, with toxin contents of 221–845 µg MC-LR equivalents/g DW and 10–18 µg ATX equivalents/g DW measured in mat samples taken in 2001 [57]. These mats were also linked to the death of lesser flamingos. MC variants detected in hot springs and in tissues from lesser flamingos dead nearby included MC-LR MC-RR MC-LF and MC-YR [57]. MCs were also detected in a series of hot springs (Al Khawbah, 70 °C; Bani Malik, 48 °C; and Al Bozah, 52 °C) located in Jazan, Saudi Arabia, which were widely used for bathing and recreation by local citizens and tourists [58]. Mats contained 468–512 μg eq. MCLR/g DW whereas water concentrations reached 5.7 µg/L. Two MC-producing strains (*Oscillatoria limosa* and *Synechococcus lividus*) were isolated from the mats and their production of MC-LR and MC-RR was confirmed by HPLC in culture biomass.

Moreira et al. [78] screened hot springs on Azores Islands, Portugal (Caldeira das Furnas, Caldeira Vella, Furnas Hot Springs; water temperatures of 30–54 °C) for the presence of genes involved in the biosynthesis of MC CYN and PST. The gene *mcyA* was detected in all springs, but the simultaneous presence of *mcyA* and *mcyE* was only detected in Furnas Hot Spring (40 °C). Mats from Furnas also contained the two CYN-biosynthesis genes screened (*cyrB* and *cyrC*), while no sxt gene was detected in any of the hot springs. Despite these interesting findings, the study does not report results regarding the sequences identity or the detection of cyanotoxins.

Beyond the species mentioned, hot spring mats are known to contain filamentous stigonematalean cyanobacteria such as the genera *Mastigocladus* and its phylogenetically close neighbor *Fischerella* [74]. MC-production has been confirmed in *Fischerella* and *Hapalosiphon* strains from tropical freshwater springs and soils [79,80,81]. Furthermore, whole genome analyses of *Fischerella* sp. PCC9509 have revealed that more than 5% of its genes encode enzymes (nonribosomal peptide synthases, and polyketide synthases) potentially involved in the synthesis of cyanotoxins and other secondary metabolites [81]. In this sense, thermophilic Stigonematales pose a reasonable target to look into by future cyanotoxicity research works in geothermal habitats.

## 8. Concluding Remarks

This review brings to the forefront the presence of cyanotoxins in a variety of extreme ecosystems (polar deserts, hot deserts, hypersaline environments, alkaline lakes and hot springs). The toxins detected include neurotoxins –the alkaloids ATX, ATX (S) and PSTs; the amino acids, AEG, BMAA and DAB- and hepatotoxins –CYN, MCs, NOD-. Within a general context of scarce and patchy information, most cyanotoxin reports available concern polar ecosystems and alkaline lakes. As far as we know, the only strains isolated and hence unequivocally confirmed as cyanotoxin producers in extreme habitats belong to *Arthrospira* spp. from alkaline lakes in the African Rift Valley, and *Oscillatoria* and *Synechococcus* from hot springs in Saudi Arabia. The remaining proposed producers remain just putative and comprise of about 8 additional genera (Table 2 and Table 3): *Anabaena*, *Lyngbya*, *Nostoc*, *Oscillatoria*, *Phormidium*, *Planktothrix*, *Scytonema* in the poles; *Anabaena* and *Microcoleus*/*Phormidium* in hot deserts; and *Phormidium* in hot springs.

Current background on this topic is really valuable and provides solid data that cyanotoxins are a worldwide phenomenon. However, the amount of information of cyanotoxicity in extreme environments is still very far from that available for temperate freshwaters dominated by planktonic taxa. Keeping this in mind, in this last section we will summarize some of the main knowledge gaps that need to be filled toward an initial risk assessment of cyanotoxins in these amazing and extreme habitats.

### 8.1. Challenges and Knowledge Gaps

Research in extreme ecosystems is not an easy task at all. The harsh environmental conditions result in sampling campaigns which are much more problematic than those performed under mild conditions. Furthermore, the organisms from extreme habitats are often difficult to isolate and transport alive to the laboratory. Also, the specific environmental conditions/microhabitats in which they thrive cannot be easily simulated in the laboratory. This drives to one of the main issues faced in this topic: the almost complete lack of cyanotoxins-producing cultures isolated from the ecosystems mentioned, with the exceptions of five strains worldwide (Table 3).

This lack of isolates precludes from unequivocally confirming the taxonomic adscription of cyanotoxin producers, and/or from quantifying the cell quotas or performing regulation experiments under controlled conditions. It is true that part of these problems can be overcome by the recommendable use of non-culturing dependent techniques, which on the other hand would provide a much more real overview of the system. The use of in situ –omics (metagenomic and meta-transcriptomic) combined with detailed physicho-chemical parameters and comprehensive statistics would (and must) definitely help in trying to identify some of the key candidates for cyanotoxin production and their main environmental drivers. However, it would be still difficult to evaluate the quantitative aspects of cyanotoxin production (cell quotas, percentage of extracellular share of the toxin) without cultures. In this sense, an important part of the efforts of researchers should be driven to obtaining viable cultures of toxic cyanobacteria from extreme environments, improving the isolation and culturing techniques, so that they can be subsequently subjected to studies in the fashion of those in Table 1 of this review.

Regarding the above mentioned in situ –omics, it is striking the scarcity of their use in the cyanotoxicity studies from extreme environments. Metagenomics focused on cyanotoxins producers are limited to results by Kleinteich and co-workers on CYN-producers in the Antarctica [31]. About transcriptomics, there is only one qualitative study detecting mRNA of the PST-synthesis gene *sxtA* in [33] but no data are available on quantitative transcriptomics/gene expression levels of any cyanotoxin biosynthesis gene. Studies focusing on proteomics of toxin genes are absent too, and there is no characterization of the activity of toxin-biosynthesis enzymes from these systems. This last aspect could be very relevant as hypothetically, the toxin biosynthesis enzymes (nonribosomal peptide synthetases, polyketide synthases, amidinotransferases etc.) from organisms of these extreme environments may differ in their tolerance ranges from those of mild conditions, and therefore they might be of great interest for biotechnological purposes.

Following the environmental limits of cyanotoxin biosynthesis, many of the key factors varying in extreme systems have not been studied yet for cyanotoxin production, neither in culture nor in situ. Indeed, the information from any kind of culture is mostly focused on variations of temperature, light irradiance, pH and salinity (Table 1). Some of the key stressors lacking in those studies are cycles of desiccation-hydration and/or freezing-thawing, UV radiation, prolonged darkness (e.g., as such happening in winter in polar zones), availability of ions directly or indirectly affected by pH—such as CO_3_^2−^, Iron, Magnesium, Calcium etc.—just to mention a few.

Another aspect to point out is the lack of geographically extensive studies in extreme ecosystems. In spite of the effort already made in work summarized in this review, they cover an insignificant proportion of the immense area of some of these habitats, especially the vast polar deserts. There is only one study covering a significant number of water bodies, namely that by Trout-Haney et al. [40] in 18 Greenland Lakes. For social and economic reasons, ecosystems from temperate freshwaters have received much more attention, and yet extensive studies are not as abundant as they should, there are some good examples in the USA and in Europe. A remarkable effort was made by the U.S. Environmental Protection Agency (EPA), who monitored 1161 lakes for cyanotoxins in USA as part of the EPA National Lakes Assessment 2007 [41]. In Europe, some of the most extensive work covered 21 lakes in Germany [82], 65 lakes in Serbia [83], 94 lakes and reservoirs in Czech Republic [84], or 14 waterbodies in Spain with 31 potentially toxic strains isolated [85]. The information for European freshwaters is expected to keep increasing in the following years driven by legislative obligations such as the Directive 2006/7/EC concerning the management of bathing water quality [86]. Although it is difficult that studies in extreme ecosystems reach these magnitudes, given that they are often located in remote areas not affected by legislation and consequent investments, at least it would be desirable to make a research effort to increase their geographical extension to make them as much representative as practicable. 

Moving one step further, it is well known that cyanobacteria can thrive not just in ecosystems mentioned so far (poles, hot deserts, hypersaline environments, alkaline lakes, hot springs) but also in a number of further extreme environments. For instance, rocks pose an interesting biotope where cyanobacteria are present as endolithic populations [87], or caves, characterized by an unusually low irradiation, such as the 0.01–0.02 µmol photons m^−2^·s^−1^ under which *Hapalosiphon* and *Gloeocapsa* can thrive [87,88]. To our knowledge, no studies have focused on seeking cyanotoxins in these systems, or at least there are no reports of their presence so far. Even more, this disentanglement of cyanotoxin production at the edge of life could be extended beyond Earth itself, e.g., in Mars simulated conditions or space flights where there are actual experiences of cyanobacterial survival [61].

### 8.2. Indications for Risk Assessment

From the knowledge summarized in this review, it is evident that the information gaps are too many to allow proper ecological or health risk assessments of cyanotoxins in most extreme environments. However, in this section, we will point out some first recommendations toward this purpose.

In principle, we may think that the risks of cyanotoxins presence in these extreme areas are much lower than those in ecosystems under mild conditions. We could argue this both from the point of the quantitative production of toxins—expected to be lower in extreme systems because of the less usual development of mass proliferations—as well as from the apparently reduced exposure of ecosystem organisms—extreme areas are in general expected to be less biodiverse than those in mild conditions; and they often coincide with areas with a very low human population density. This being reasonable, we still believe that the scientific community or the policy makers cannot just ignore or overlook the risks in extreme ecosystems, since there are some factors that could generate spatially localized (but actual) risks.

One of the factors affecting ecological risks is a possible bioaccumulation of cyanotoxins in the trophic chain. Bioaccumulation is relatively well described for freshwaters from temperate latitudes [89] but there are almost no data from extreme environments with the exception of MC bioaccumulation in lesser flamingos from Africa [63,90]. Other aspect that may have a yet unknown influence is climate change/global warming that could affect the composition of microbial communities (including cyanobacteria) of all ecosystems but especially of polar zones and deserts. Some of its possible effects may include an expansion of current deserts toward adjacent areas, and in polar zones the deglaciation with the consequent colonization of new areas, or the spreading of some microbes from temperate zones toward these cold zones. Regarding cyanobacteria, some of the considered as “invasive” species are indeed toxic, such as the CYN producers *Cylindrospermopsis raciborskii* and *Chrysosporum/Aphanizomenon ovalisporum* [91]. Therefore, whether toxic cyanobacteria could increase their abundance in extreme systems in response to climate change trends is one of the key aspects to disentangle by research in the following years.

Regarding human health risk assessment, an aspect to consider is that exposed populations could be more abundant than one could initially assume, including: (a) natives in close contact with the natural environments (e.g., the Inuit in the Arctic), which in some areas even use the cyanobacterial biomass itself as food supplement (e.g., African population use *Arthrospira* biomass collected from certain lakes) [92]; (b) professionals like the Army working in hot deserts [50,51] or in Antarctica, and scientists/researchers specially in polar bases like those in the Antarctica; (c) tourists, which are increasing their number in the last years toward unusual destinations such as hot spring areas and even to the Antarctica. In spite of this, the insufficient information has resulted in an almost complete lack of risk assessment efforts in extreme environments. The notable exception is the work by Metcalf et al. based on their detection of MCs in cyanobacterial crusts from Qatar desert [50]. These authors calculated that the dose of MCs in cyanobacterial crusts from Qatar could exceed a Tolerable Daily Intake (TDI) value of 1–2 ng kg^−^^1^ day^−^^1^ for an average adult, taking into account NOAEL (No Observed Adverse Effect Level) and LOAEL (Lowest Observed Adverse Effect Level) values via nasal spray inhalation of purified MC-LR in aqueous solution and the amount of dust inhaled by a person from these dried crusts.

Hopefully, the fulfilment of the research gaps summarized here, together with others arising on the way, will enable researchers to better understand the driving forces of cyanotoxicity in extreme environments, as well as to present an actual dimension to its consequences for ecosystems and human health.

## Figures and Tables

**Table 1:** Summary of environmental factor ranges tested on cyanotoxin production by cyanobacterial cultures. Only the key factors varying in extreme environments are included. Range refers to the overall of all studies, yet each individual genus may not have been exposed to the entire range. Sources: [11,12,13,14,15,16] and references therein.

Toxin	Environmental Factor	Range Assayed	Genera
Anatoxin-*a*	Temperature (°C)	10–30	*Anabaena, Aphanizomenon, Cuspidothrix* (basionym *Aphanizomenon*), *Phormidium*
	Light irradiance (µmol photons m^−2^·s^−1^)	2–128	*Anabaena, Aphanizomenon, Cuspidothrix* (basionym *Aphanizomenon*), *Phormidium*
Cylindrospermopsin	Temperature (°C)	15–35	*Aphanizomenon, Chrysosporum,* (basionym *Aphanizomenon*)*, Cylindrospermopsis, Oscillatoria*
	Light irradiance (µmol photons m^−2^·s^−1^)	2–340	*Aphanizomenon, Chrysosporum (*basionym *Aphanizomenon), Cylindrospermopsis, Oscillatoria*
Microcystins	Temperature (°C)	10–35	*Anabaena, Microcystis, Nostoc, Planktothrix*
	Light irradiance (µmol photons m^−2^·s^−1^)	1–205	*Anabaena, Microcystis, Nostoc, Planktothrix*
	pH	7–14	*Microcystis*
	Salinity (‰)	0–10	*Microcystis*
Paralytic Shellfish Toxins	Temperature (°C)	15–28	*Anabaena, Aphanizomenon, Cuspidothrix* (basionym *Aphanizomenon*)*, Cylindrospermopsis, Raphidiopsis*
	Light irradiance (µmol photons m^−2^·s^−1^)	15–150	*Anabaena, Aphanizomenon, Cylindrospermopsis, Raphidiopsis*
	pH	7–9.5	*Anabaena, Cylindrospermopsis, Raphidiopsis*
	Salinity (‰)	0–10	*Anabaena, Cylindrospermopsis, Raphidiopsis*
Nodularin	Temperature (°C)	10–30	*Nodularia*
	Light irradiance (µmol photons m^−2^·s^−1^)	2–155	*Nodularia*
	Salinity (‰)	0–35	*Nodularia*

**Table 2:** Reports of cyanotoxins and cyanotoxin biosynthesis gene detection in field samples from extreme environments I: polar deserts. Abbreviations: DW, dry weight; nr, not reported; NRPS, nonribosomal peptide synthetase; PKS, polyketide synthase. Toxins: ATX, anatoxin-a; CYN, cylindrospermopsin; MC, microcystins; NOD, nodularin; PST: paralytic shellfish toxins.

Ecosystem	Toxins	Location	Sample Type	Potential Producers ^1^	Toxin Content (µg/g DW)	Toxin Genes	References
Polar deserts (Artic)	ATX	Svalbard, Norway	Biocrusts	*Oscillatoria* spp., *Phormidium* spp.	0.3–0.6	nr	[37]
	MC	Baffin Island, Canada	Mats	*Nostoc* sp., *Phormidum* spp., *Planktothrix* sp.	<0.02–4.3	*mcyA*, *mcyB*, *mcyE*	[32,33]
		Svalbard, Norway	Biocrusts	*Nostoc* spp., *Oscillatoria* spp., *Phormidium* spp.	0.1–11.1	nr	[37]
			Cyanolichens	*Nostoc* spp.	nr	*mcyE*	[38,39]
		Lapland, Finland	Cyanolichens	*Nostoc* spp.	nr - <10	*mcyE*	[38,39]
		18 lakes in SW Greenland	Whole water sample	nr	0.005–0.4 µg/L	nr	[40]
	PST	Baffin Island, Canada	Mats	*Scytonema* sp., *Lyngbya* sp.	0.02	*sxtA, sxtA mRNA*	[33]
Polar deserts (Antarctica)	CYN	Adelaide Island	Mats	*Oscillatoria* sp.	<0.01–0.16 ^1^	*cyrA*, *cyrB*, *cyrJ*	[31]
	MC	Adelaide Island	Mats	*Nostoc* sp.	0.01–0.30 ^1^	*mcyA*, *mcyE*	[31]
		Mc Murdo Ice Shelf	Mats	nr	nr	nr	[34]
			Mats	*Phormidium*, *Oscillatoria*, *Nostoc*, *Anabaena*	11.4	NRPS, PKS	[35]
			Mats	*Nostoc* sp.	<0.001–15.9	*mcyE*	[36]
		Livingston Island	Mats	*Nostoc* sp., *Phormidum* spp., *Planktothrix* sp.	<0.01–1.7	nr	[32]
	NOD	Mc Murdo Ice Shelf	Mats	nr	nr	nr	[34]

^1^ Toxin content measured as µg toxin/g organic mass.

**Table 3:** Reports of cyanotoxins and cyanotoxin biosynthesis gene detection in field samples from extreme environments II (hot deserts, alkaline lakes and hot springs). Abbreviations: DW, dry weight; nr, not reported. Toxin abbreviations: AEG, N-(2-aminoethyl) glycine; ATX, anatoxin-a; ATX (S), anatoxin-a (s); BMAA, b-N-methylamino-l-alanine; DAB, 2,4-diaminobutyric acid; MC, microcystins; NOD, nodularin.

Ecosystem	Toxins	Location	Sample Type	Potential Producers ^1^	Toxin Content (µg/g DW)	Toxin Genes	References
Hot deserts	BMAA	Qatar desert	Biocrusts	nr	nr	nr	[49,51]
		Gobi Desert, Mongolia	Phytoplankton (springs)	*Anabaena* spp.	nr	nr	[52]
	DAB, AEG	Qatar desert	Biocrusts	nr	DAB: 2.8–7.0 AEG: 0.7–4.4	nr	[53]
		Gobi Desert, Mongolia	Phytoplankton (springs)	*Anabaena* spp.	nr	nr	[52]
	MC	Qatar desert	Biocrusts	nr	0.001–0.05	*mcyD*	[50]
	ATX(S)	Qatar desert	Biocrusts	*Microcoleus/Phormidum* spp.	0.001 ^2^	nr	[50]
Alkaline lakes	ATX	4 lakes in Rift Valley, Kenya	Phytoplankton	*Arthrospira fusiformis*	0.3–223	nr	[54,55]
	MC	4 lakes in Rift Valley, Kenya	Phytoplankton	*Arthrospira fusiformis*	1.6–4593	nr	[54,55]
	NOD	Lake Burdur, Turkey	Water	*Nodularia spumigena*	0.30–4.82 µg/L	nr	[56]
Hot springs	ATX	Lake Bogoria, Kenya	Mats	*Phormidium, Oscillatoria*, *Synechococcus*	10–18	nr	[57]
	MC	Lake Bogoria, Kenya	Mats	*Phormidium, Oscillatoria*, *Synechococcus*	221–845	nr	[57]
		Jazan, Saudi Arabia	Mats	*Oscillatoria limosa Synechococcus lividus*	468–512.5	nr	[58]
			Water	nr	4.6–5.7 µg/L	nr	[58]

^1^ Potential producers isolated and confirmed as toxin producers are underlined and marked in bold; ^2^ Toxin content measured as µg Neostigmine equivalents/g dry weight.
